# Cofactor-independent C–C bond cleavage reactions catalyzed by the AlpJ family of oxygenases in atypical angucycline biosynthesis

**DOI:** 10.3762/bjoc.20.102

**Published:** 2024-05-23

**Authors:** Jinmin Gao, Liyuan Li, Shijie Shen, Guomin Ai, Bin Wang, Fang Guo, Tongjian Yang, Hui Han, Zhengren Xu, Guohui Pan, Keqiang Fan

**Affiliations:** 1 State Key Laboratory of Microbial Resources, Institute of Microbiology, Chinese Academy of Sciences, No. 1 Beichen West Road, Beijing 100101, China; 2 University of Chinese Academy of Sciences, No. 1 Yanqihu East Road, Beijing 101408, China; 3 State Key Laboratory of Natural and Biomimetic Drugs, School of Pharmaceutical Sciences, Peking University, 38 Xueyuan Road, Beijing 100191, China

**Keywords:** angucyclines, aromatic polyketide, biosynthesis, cofactor-independent oxygenase, oxidative rearrangement reaction

## Abstract

Biosynthesis of atypical angucyclines involves unique oxidative B-ring cleavage and rearrangement reactions, which are catalyzed by AlpJ-family oxygenases, including AlpJ, JadG, and GilOII. Prior investigations established the essential requirement for FADH_2_/FMNH_2_ as cofactors when utilizing the quinone intermediate dehydrorabelomycin as a substrate. In this study, we unveil a previously unrecognized facet of these enzymes as cofactor-independent oxygenases when employing the hydroquinone intermediate CR1 as a substrate. The enzymes autonomously drive oxidative ring cleavage and rearrangement reactions of CR1, yielding products identical to those observed in cofactor-dependent reactions of AlpJ-family oxygenases. Furthermore, the AlpJ- and JadG-catalyzed reactions of CR1 could be quenched by superoxide dismutase, supporting a catalytic mechanism wherein the substrate CR1 reductively activates molecular oxygen, generating a substrate radical and the superoxide anion O_2_^•−^. Our findings illuminate a substrate-controlled catalytic mechanism of AlpJ-family oxygenases, expanding the realm of cofactor-independent oxygenases. Notably, AlpJ-family oxygenases stand as a pioneering example of enzymes capable of catalyzing oxidative reactions in either an FADH_2_/FMNH_2_-dependent or cofactor-independent manner.

## Introduction

The angucyclines represent a large class of natural products biosynthesized by type II polyketide synthases. Within this category, a distinctive subset of compounds, including jadomycin, gilvocarcin, kinamycin, fluostatin, and lomaiviticin, arises from typical angucycline intermediates via oxidative C–C bond cleavage and subsequent ring rearrangement reactions. These atypical angucyclines exhibit intriguing chemical structures and various biological activities [1–2]. Notably, kinamycins demonstrate both antibacterial and antitumor activities, while lomaiviticin emerges as a potent antitumor agent, displaying remarkable IC_50_ values ranging from 0.007–72 nM against 25 cultured human cancer cell lines [3–7].

**Scheme 1:**
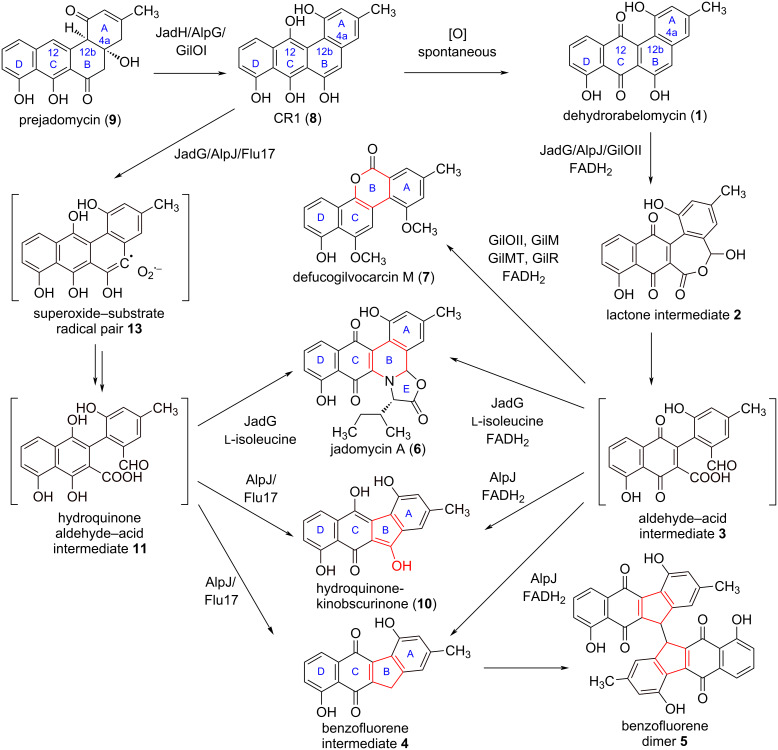
Ring cleavage and ring rearrangement reactions in the biosynthesis of atypical angucyclines.

The pivotal B-ring cleavage and rearrangement in atypical angucycline biosynthesis are catalyzed by the AlpJ-family oxygenases, encompassing AlpJ, JadG, and GilOII. Biochemical analyses reveal the capability to transform the shared angucycline intermediate, dehydrorabelomycin (DHR, **1**), into products with unique chemical scaffolds (Scheme 1) [1–2,8–14]. Noteworthy is the prerequisite for FADH_2_/FMNH_2_, provided by *E. coli* Fre or flavin reductases in corresponding biosynthetic pathways, to facilitate these oxidation reactions. Experimental data support that **1** was firstly oxidized to a lactone intermediate **2** via Baeyer–Villiger oxidation, followed by hydrolysis to yield another crucial aldehyde/acid intermediate **3** [11,15]. Commencing from **3**, diverse ring rearrangement reactions can occur, leading to the formation of distinct products.

In the AlpJ-catalyzed reaction, compound **3** undergoes ring contraction reactions, yielding a benzofluorene intermediate **4** and the dimer **5**, both featuring a kinamycin skeleton (Scheme 1) [11–12]. Recent investigations unveiled the catalytic activity of the O-methyltransferase-like protein AlpH, which catalyzes a unique SAM-independent coupling of ʟ-glutamylhydrazine and **3** through a rare Mannich reaction. This results in the formation of prekinamycin harboring a diazo group [14]. Given the presence of AlpJ-like oxygenases and ʟ-glutamylhydrazine biosynthetic enzymes in the gene clusters of lomaiviticin, nenestatin, and fluostatins [16–20], it is plausible that the corresponding biosynthesis involves analogous oxidative ring opening and rearrangement steps. On the other side, in JadG-catalyzed reactions, compound **3** reacts with ʟ-isoleucine or other amino acids, leading to the formation of jadomycin A (**6**) or analogues, respectively [10]. In the gilvocarcin biosynthesis, the conversion of **3** to defucogilvocarcin M (**7**), featuring a benzo[*d*]naphtho[1,2-*b*]pyran-6-one backbone, necessitates the collaborative actions of GilOII and three additional tailoring enzymes, namely GilM, GilMT, and GilR [9].

To date, all available biochemical data consistently demonstrate the FADH_2_/FMNH_2_-dependent nature of AlpJ-family oxygenases when utilizing **1** as the substrate. Intriguingly, these enzymes exhibit notable similarities to cofactor-independent anthrone oxygenases, exemplified by TcmH and ActVA-Orf6, in both protein sequences and structures (see Figure S1, Supporting Information File 1) [21–22]. In this study, we reveal the previously undisclosed facet that AlpJ-family oxygenases can function as cofactor-independent oxygenases when the hydroquinone intermediate CR1 (**8**) serves as the substrate. In this context, the enzymes autonomously catalyze oxidative C–C bond cleavage, ring opening, and rearrangement reactions, yielding the respective products. Furthermore, the reactions of **8** catalyzed by JadG and AlpJ could be quenched by superoxide dismutase (SOD), supporting a catalytic mechanism involving the generation of a substrate radical and the superoxide anion O_2_^•−^. This discovery sheds more light on the biosynthesis of atypical angucyclines and extends the repertoire of cofactor-independent oxygenases.

## Results and Discussion

### Cofactor-independent oxidative ring cleavage and rearrangement reactions catalyzed by AlpJ with CR1 (**8**) as substrate

Given the resemblance of AlpJ-family oxygenases to cofactor-independent anthrone oxygenases rather than to the conventional FADH_2_-dependent counterparts, we aimed to investigate the potential for catalyzing oxidation reactions in a cofactor-independent manner. For cofactor-independent oxygenases such as TcmH and ActVA-Orf6, the substrates were able to reductively activate molecular oxygen, thereby enabling subsequent oxidation reactions [21–24]. However, in the documented reactions of AlpJ-family oxygenases, the substrate **1** appeared insufficient in providing the requisite reducing power, considering the quinone structure. Consequently, we redirected our focus towards alternative, more electron-rich substrates. In a preceding investigation, we identified that the bifunctional enzyme JadH proficiently converted prejadomycin (**9**) to **8**, a compound demonstrated to spontaneously oxidize to **1** under aerobic conditions [25]. This observation not only affirmed **8** as an intermediate in jadomycin biosynthesis but also suggested a role as a more electron-rich substrate with the potential for direct activation of molecular oxygen.

**Figure 1:**
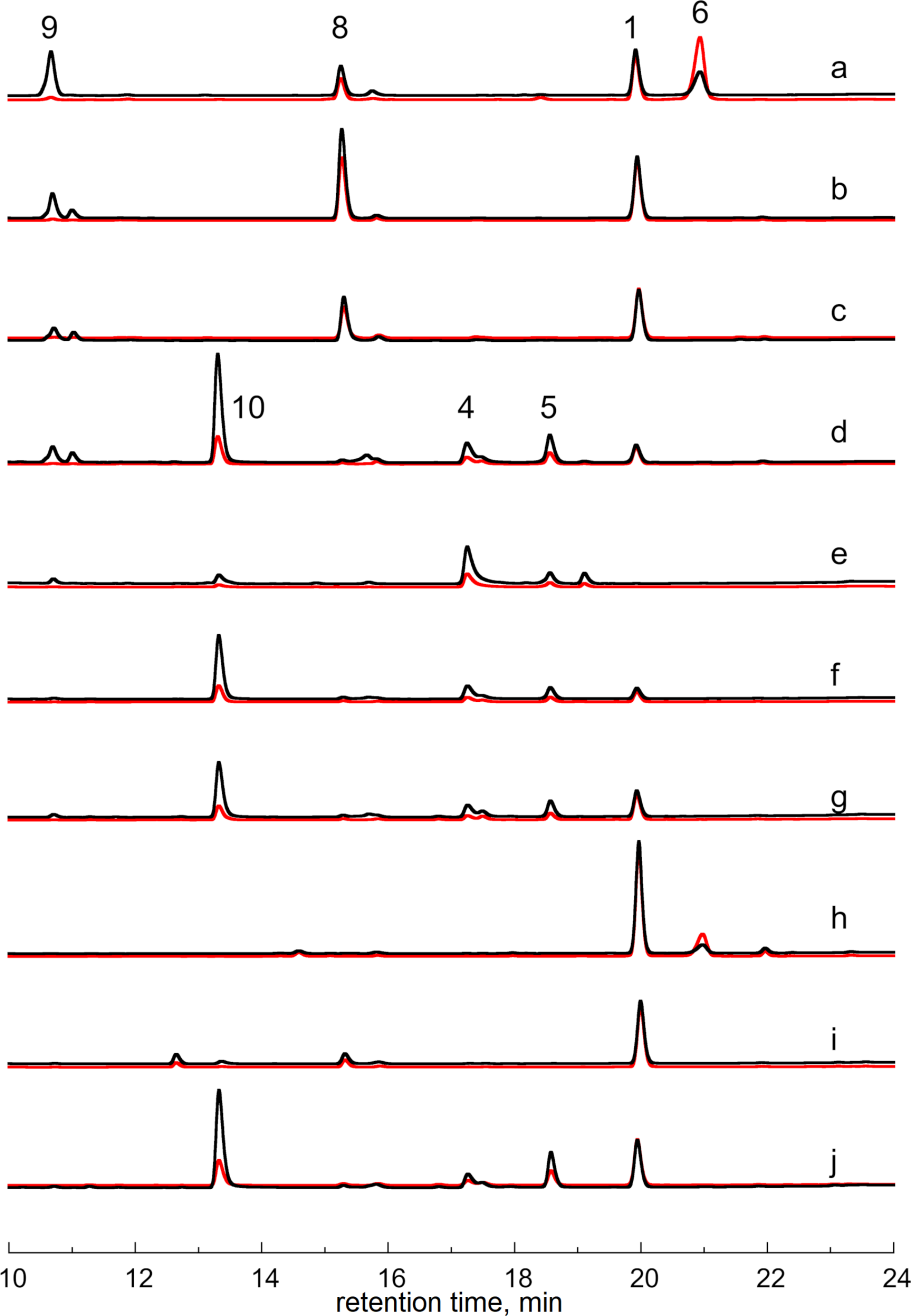
HPLC traces of reaction mixtures of AlpG, AlpJ, Flu17, and JadG. (a) standards of prejadomycin (**9**), CR1 (**8**), dehydrorabelomycin (**1**), and jadomycin A (**6**); (b) **9** + AlpG + NADPH + FAD; (c) **9** + JadH + NADPH + FAD; (d) **9** + AlpG + AlpJ + NADPH + FAD; (e) **1** + AlpJ + Fre + NADPH + FAD; (f) **8** + AlpJ; (g) **8** + Flu17; (h) **8** + JadG + ʟ-isoleucine; (i) **8** + JadG; (j) **8** + AlpJ + ʟ-isoleucine. UV absorptions at 266 nm (black line) and 313 nm (red line) are displayed for each reaction.

We first confirmed the generation of **8** in the biosynthetic pathway of kinamycin. The analysis of the *alp* gene cluster from *Streptomyces ambofaciens* revealed AlpG as the homolog of JadH. The N-terminal His_6_-tagged construct of AlpG was expressed and purified to homogeneity in *E. coli* (Figure S2, Supporting Information File 1). The purified AlpG displayed a light yellow color, and the associated prosthetic group was identified as FAD through HPLC analysis of the denatured AlpG supernatant (Figure S3, Supporting Information File 1). As anticipated, AlpG exhibited the capacity to convert **9** to **8** and **1** in the presence of NADPH and FAD (Figure 1, trace b). This result underscores **8** as a biosynthetic intermediate in kinamycin biosynthesis, establishing AlpG as a bifunctional hydroxylase/dehydratase same to JadH (Figure 1, trace c) [25].

Next, employing the AlpG-catalyzed reaction of **9** as an in situ generation system of **8**, we introduced AlpJ (in the absence of Fre) to the reaction. Apart from the spontaneous formation of a limited quantity of **1** derived from **8**, we detected the generation of products, including the previously characterized benzofluorene intermediate **4** and benzofluorene dimer **5**, alongside a novel compound, designated as **10** (Figure 1, trace d). High-resolution mass spectrometry (HRMS) analysis of **10** ([M − H]^−^ calcd for C_18_H_12_O_5_, 307.0612; found, 307.0607, Figure S4, Supporting Information File 1) suggested a possible identity as hydroquinone–kinobscurinone [11]. However, attempts to elucidate the chemical structure of **10** were not successful due to the inherent instability. These findings indicate that AlpJ, in the absence of FADH_2_, is capable of catalyzing the ring cleavage and contraction reactions of **8**. However, it cannot be definitively ruled out that AlpG in the reaction might contribute trace amounts of FADH_2_ to AlpJ.

Subsequently, we carefully isolated **8** from a scaled-up reaction of AlpG with **9** as the substrate [25]. When **8** was incubated with AlpJ in the absence of any cofactors, the formation of **4**, **5**, and **10** was observed (Figure 1, trace f). Additionally, we conducted a reaction using **1** and AlpJ in the presence of FADH_2_ generated by Fre. As anticipated, compounds **4** and **5** were detected, and notably, a small amount of **10** was also observed (Figure 1, trace e). As expected, in the negative control reactions, **8** was spontaneously oxidized to **1** without any enzyme added, and no **4**, **5**, or **10** was observed; **1** remained unchanged in the reaction mixture with Fre, NADH, and FAD but without AlpJ (Figure S5, Supporting Information File 1). These results demonstrate that AlpJ does not necessitate any cofactors for catalyzing the oxidation reactions of **8**, yielding products identical to those observed in the FADH_2_-dependent reactions of AlpJ using **1** as the substrate.

### Cofactor-independent oxidative ring cleavage and rearrangement reactions of CR1 (**8**) catalyzed by homologous proteins of AlpJ

To extend the generality of our findings, we assessed the cofactor-independent catalytic capabilities of other homologous proteins of AlpJ. Previous investigations indicated that the biosynthesis of fluostatins involves analogous B-ring cleavage and contraction steps as observed in kinamycin biosynthesis [2,13,19,26]. A sequence analysis of a reported fluostatin biosynthetic gene cluster in reassembled environmental DNA identified Flu17 as the putative ring opening oxygenase [8]. The N-terminal His_6_-tagged Flu17 was expressed and purified to homogeneity in *E. coli* BL21 (Figure S2, Supporting Information File 1). Significantly, Flu17 alone demonstrated the ability to convert **8** into products **4**, **5**, and **10** (Figure 1, trace g). Furthermore, incubating **8** with JadG and ʟ-isoleucine yielded **6** (Figure 1, trace h). Consistent with expectations, omission of ʟ-isoleucine from the reaction resulted in the absence of detectable **6** (Figure 1, trace i). The addition of ʟ-isoleucine to the reaction of AlpJ and **8** did not alter the product profile (Figure 1, trace j).

Our findings demonstrate the capacity of AlpJ-family oxygenases to catalyze the ring cleavage and rearrangement reactions of **8** in a cofactor-independent manner. Moreover, these enzymes efficiently convert **8** into products featuring distinct chemical skeletons, aligning with outcomes observed when catalyzing the oxidation of **1** with FADH_2_ as a cofactor [10–13]. The parallel behavior of Flu17 and AlpJ in the reaction with **8** provides additional support for the shared oxidative B-ring cleavage and contraction steps in the biosynthesis of fluostatins and kinamycins. Nevertheless, the mechanism by which a B-ring-contracted intermediate with a benzo[*b*]fluorene skeleton undergoes further conversion into fluostatins with a benzo[*a*]fluorene skeleton remains to be elucidated [27–28].

### Inhibition of cofactor-independent catalytic activities of AlpJ-family oxygenases by superoxide dismutase

There are many reported cofactor-independent oxygenases, including anthrone oxygenases such as ActVA-Orf6 and nogalamycin monooxygenase (NMO or SnoaB) [21,23,29–32], TnmJ and TnmK2 [24], 1-*H*-3-hydroxy-4-oxoquinaldine-2,4-dioxygenase (HOD) [33–34], and urate oxidase [35–36]. Investigations into NMO and HOD revealed the utilization of substrates to activate molecular oxygen, leading to the generation of a substrate radical and the superoxide anion O_2_^•−^ [29,33,37]. The well-established superoxide trapping agent superoxide dismutase (SOD) has demonstrated significant inhibition of the NMO-catalyzed monooxygenation reaction [29].

**Figure 2:**
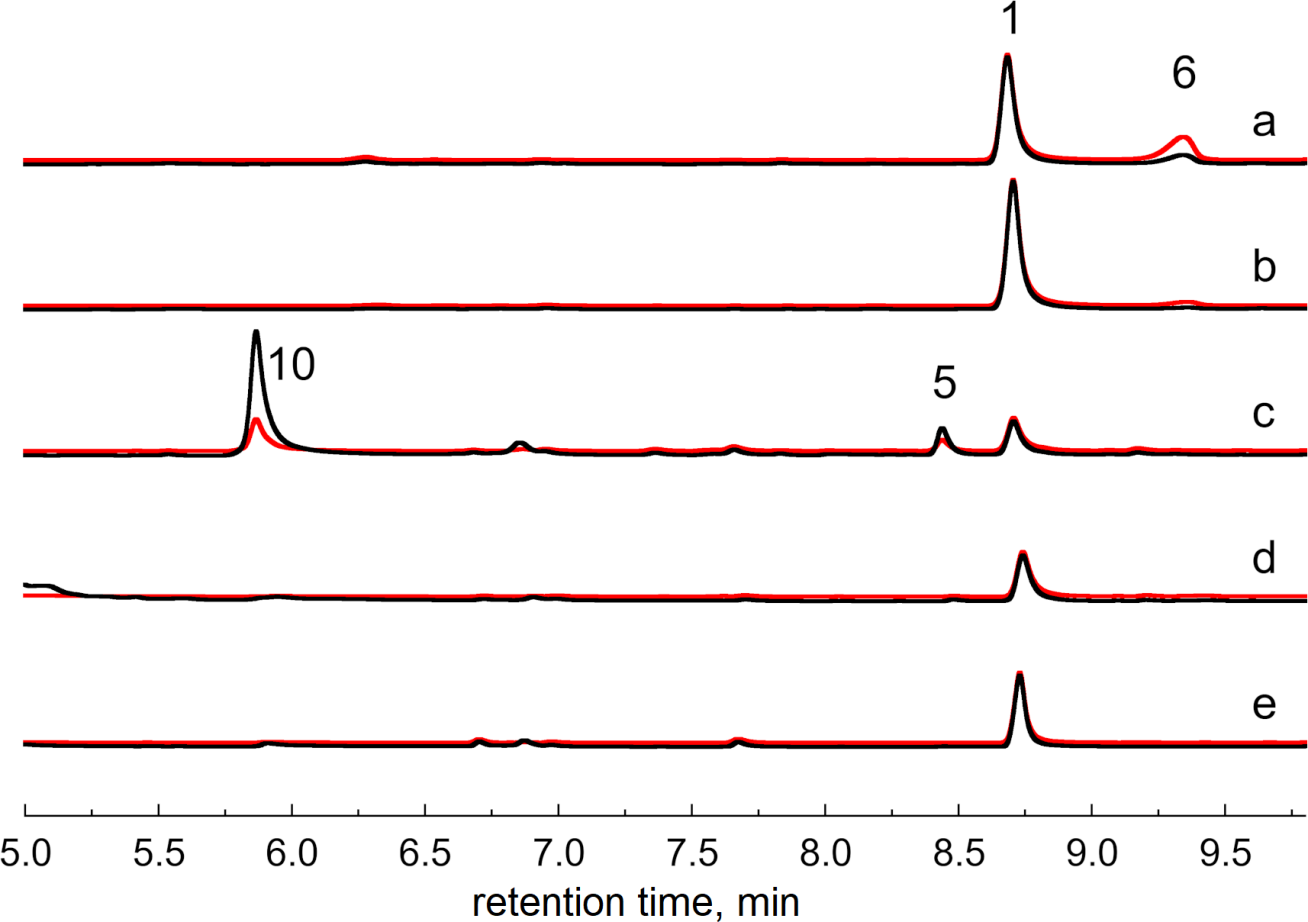
HPLC traces of reactions of JadG, AlpJ, or Flu17 quenched by SOD. (a) **8** + JadG + ʟ-isoleucine; (b) **8** + JadG + ʟ-isoleucine + SOD (5,000 U/mL); (c) **8** + AlpJ; (d) **8** + AlpJ + SOD (5,000 U/mL); (e) **8** + Flu17 + SOD (5,000 U/mL). UV absorption at 266 nm (black line) and 313 nm (red line) are displayed for each reaction.

To probe the potential involvement of a radical-mediated catalytic mechanism in AlpJ-family oxygenases, we investigated the impact of supplementing the reaction mixture of **8** and AlpJ, Flu17, or JadG with SOD. The results revealed that adding 5,000 U/mL SOD quenched the tested reactions, with no discernible products detected for AlpJ, Flu17, or JadG (Figure 2). Furthermore, a dose-dependent inhibition of the JadG-catalyzed reaction by SOD was observed (Figure S6, Supporting Information File 1). These findings support the presence of the superoxide anion O_2_^•−^ and underscore the indispensable role in the reactions catalyzed by AlpJ-family oxygenases with **8**.

### Proposed catalytic mechanism of cofactor-independent AlpJ-family oxygenases

**Scheme 2:**
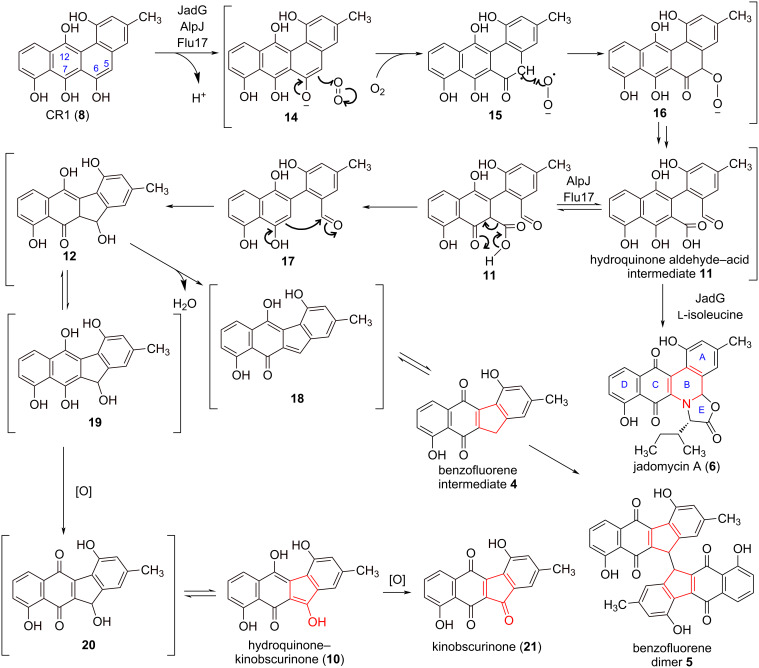
Proposed catalytic mechanism of cofactor-independent AlpJ-family oxygenases.

The class of cofactor-independent oxygenases presents a captivating group of enzymes that harness substrates to directly activate molecular oxygen. A recent investigation into the cofactor-independent enzyme NMO, employing dithranol as the substrate, unveiled the presence of a dithranyl radical and superoxide anion pair [29]. The production of the superoxide anion was captured using 1-hydroxy-3-methoxycarbonyl-2,2,5,5-tetramethylpyrrolidine (CMH) and quantified by detecting the stable CM^•^ radical through electron paramagnetic resonance. Based on previous studies of cofactor-independent oxygenases and our results, we propose a radical-mediated catalytic mechanism for the cofactor-independent AlpJ-family oxygenases (Scheme 2).

The catalytic cascade was initiated by deprotonation of **8** to form a substrate anion, which activated molecular oxygen to generate a substrate radical and the superoxide anion O_2_^•−^. The ensuing radical rebound reaction led to the formation of a peroxide intermediate, which in turn underwent a ring opening reaction to generate the hydroquinone aldehyde–acid intermediate **11**. In JadG-catalyzed reactions, compound **11** participated in a reaction with ʟ-isoleucine to yield **6**. In contrast, in AlpJ- or Flu17-catalyzed reactions, **11** underwent decarboxylation and an aldol reaction, giving rise to intermediate **12**. Subsequent dehydration of **12** led to the formation of product **4**, which through a presumed radical-mediated reaction could spontaneously convert to the dimer **5**. Additionally, compound **12** had the potential to be oxidized to hydroquinone–kinobscurinone **10**, a proposed precursor of kinobscurinone.

When employing **1** as the substrate, such cofactor-independent reactions failed to occur. It is plausible that **1** lacked the capability to reductively activate molecular oxygen owing to the quinone structure. In these instances, FADH_2_/FMNH_2_ were deemed necessary as cofactors for AlpJ-family oxygenases. These findings uncover a noteworthy substrate-controlled catalytic mechanism employed by AlpJ-family oxygenases, wherein the nature of the substrate dictates whether a cofactor-dependent or -independent reaction ensues. While it is not entirely unexpected that these enzymes could exhibit cofactor-independent oxygenase activity, given our prior discovery that the monomeric structure of AlpJ shares similarities with the dimeric cofactor-independent anthrone oxygenases, such as ActVA-Orf6 [13], the structural intricacies governing the accommodation of these two distinct mechanisms warrant further investigation.

The current findings indicate that both **8** and **1** can serve as direct substrates for AlpJ-family oxygenases. However, the dominant biosynthetic pathway in vivo remains unclear. Given that **8** has been experimentally validated as the authentic product of the JadH/AlpG-catalyzed reaction, it is recognized as a bona fide intermediate in angucycline biosynthesis [25].

On the other hand, the origin of **1**, spontaneously derived from **8** under aerobic conditions, raises uncertainties about the actual production in vivo. Furthermore, the significance of the C12 hydroxy group in the hydroquinone intermediate **11** is evident in subsequent reactions, including the formation of a hemiacetal intermediate in gilvocarcin biosynthesis [9,38–39] and the AlpH-catalyzed ʟ-glutamylhydrazine adduct formation [14]. Notably, this C12 hydroxy group is absent in the quinone intermediate **3** of the cofactor-dependent pathway. Collectively, these observations suggest that the cofactor-independent pathway is likely the favored biosynthetic route in the angucycline biosynthesis process.

## Conclusion

In this study, we showed that AlpJ-family oxygenases, traditionally recognized as FADH_2_/FMNH_2_-dependent enzymes, exhibit cofactor-independent oxygenase activity when **8** is employed as the substrate. AlpJ or the homologs Flu17 and JadG autonomously catalyze the oxidative ring cleavage and rearrangement reactions of **8**, yielding products identical to those observed in FADH_2_-dependent reactions orchestrated by AlpJ-family oxygenases using **1** as the substrate. The results allowed us to propose a catalytic mechanism for cofactor-independent AlpJ-family oxygenases, wherein the substrate **8** reductively activates molecular oxygen, generating a substrate radical and the superoxide anion O_2_^•−^. These findings unveil a captivating substrate-controlled catalytic mechanism employed by AlpJ-family oxygenases. Notably, AlpJ-family oxygenases stand as a pioneering example of enzymes capable of catalyzing oxidative reactions in either an FADH_2_/FMNH_2_-dependent or -independent manner. Up to date, many oxygenases from diverse evolutionary families, featuring varied protein folds and quaternary arrangements, have been identified as cofactor-independent oxygenases [24,29,37,40–41]. Our discoveries broaden the landscape of cofactor-independent oxygenases.

## Experimental

### Materials, culture conditions, and DNA manipulations

The substrates **8** and **1** were purified from the reaction mixture of AlpG and the cultures of *S. ambofaciens* ΔΔ*alpJW*, respectively, as previously described [11,25]. *E. coli* strains were grown in lysogeny broth (LB) [42]. Restriction enzymes, T4 DNA ligase, and KOD DNA polymerase were purchased from New England BioLabs. FAD and NADPH were purchased from Sigma-Aldrich. DNA manipulation, competent cell preparation, and transformation were performed as described previously [42].

### Protein expression and purification

The *alpG* gene was amplified by PCR from *S. ambofaciens* ΔΔ*alpW* genomic DNA using primers 5’-ggaattccatATGGAAGGGACAACGGCGGACAC-3’ and 5’-cccaagcttTCAGCGGGCGGGGCCGAA-3’, digested with *Nde*I and *Hin*dIII, and cloned into pET28a to construct the expression plasmid of N-terminal His_6_-tagged AlpG. The codon-optimized *flu17* gene was synthesized by Synbio Technologies, digested with *Nde*I and *Hin*dIII, and cloned into pET28a to afford the expression plasmid of N-terminal His_6_-tagged Flu17. Both plasmids were confirmed by sequencing and transformed into *E. coli* BL21(DE3). Protein overproduction was induced with 0.1 mM isopropyl β-ᴅ-thiogalactopyranoside at 16 °C for 15 h. Cells were collected by centrifugation (8,000*g*, 15 min) at 4 °C. The pellets were resuspended in ice-cold 50 mM 3-(*N*-morpholino)propanesulfonic acid (MOPS) buffer (pH 7.5), and the cells were disrupted by ultrasonication to obtain the cell extract. Cell debris was removed by centrifugation (14,000*g*, 15 min). The proteins were purified by Ni-NTA agarose chromatography, desalted, and concentrated by centrifugation (8,000*g*, 30 min) in 10 kDa ultrafiltration tubes (Centriplus YM series, Merck Millipore). Protein concentration was quantified by the Bradford method. AlpJ, JadG, JadH, and *E. coli* flavin reductase (Fre) were overproduced and purified by Ni-NTA agarose chromatography as described previously [10–11,43–44].

### In vitro enzymatic assay

All reactions were performed in 50 mM Tris-HCl buffer at pH 8.0. For conversion of **9** by AlpG or JadH, the reaction mixtures (50 μL) consisting of 10 μM FAD, 125 μM NADPH, 100 μM **9**, and 25 μM AlpG or JadH were incubated at 30 °C for 5 min and analyzed by HPLC directly. For conversion of **8** by AlpJ or Flu17, the reaction mixtures (50 μL) consisting of 200 μM **8** and 50 μM AlpJ or Flu17 were incubated at 30 °C for 5 min and analyzed by HPLC directly. For conversion of **8** by JadG, the reaction mixtures (500 μL) consisting of 200 μM **8**, 50 mM ʟ-isoleucine, and 500 μM JadG were incubated at 30 °C for 30 min. Reactions were terminated with 2 M HCl (2 μL) and then extracted with ethyl acetate (500 μL). The extracts were dried using a miVac Duo concentrator (SP Scientific), redissolved in 20 μL ethanol, and then analyzed by HPLC.

Reaction mixtures of AlpG, AlpJ, Flu17, or JadG were analyzed on a Waters HPLC system equipped with a 1525 pump and a 2487 dual λ UV detector using an Agilent ZORBAX SB-C18 reversed-phase column (5 μm, 4.6 × 250 mm) or a SilGreen C18AB reversed-phase column (5 μm, 4.6 × 250 mm). The elution solvents were water with 0.1% (v/v) trifluoroacetic acid (solvent A) and acetonitrile with 0.1% (v/v) trifluoroacetic acid (solvent B). A 20-min linear gradient from 25% (v/v) to 100% (v/v) solvent B was used with a flow rate of 1.0 mL⋅min^−1^. Absorbance at 266 and 313 nm was monitored. For SOD quench experiments, the reaction mixtures were analyzed on an Agilent 1260 Infinity HPLC system with a diode array detector using an Agilent ZORBAX SB-C18 rapid resolution HT column (1.8 μm, 4.6 × 100 mm). A 10 min linear gradient from 20% (v/v) to 100% (v/v) solvent B was used with a flow rate of 1.5 mL⋅min^−1^. Full spectra from 190 to 640 nm were monitored.

## Supporting Information

File 1 Sequence comparison results and phylogenetic tree of AlpJ-family enzymes and anthrone oxygenases, crystal structures of AlpJ and ActVA-Orf6, SDS-PAGE of purified proteins, HPLC traces of prosthetic group identification in AlpG, negative controls of enzymatic reactions, SOD inhibition reactions, and HRMS spectrum of hydroquinone–kinobscurinone **10**.

## Data Availability

All data that supports the findings of this study is available in the published article and/or the supporting information to this article.
