# Supplementary material for: Cofactor-independent C–C bond cleavage reactions catalyzed by the AlpJ family of oxygenases in atypical angucycline biosynthesis
Source: Beilstein J Org Chem. 2024 May 23;20:1198–206. doi: 10.3762/bjoc.20.102 (PMC11181247; doi:10.3762/bjoc.20.102)
Supplement: File 1 — Sequence comparison results and phylogenetic tree of AlpJ-family enzymes and anthrone oxygenases, crystal structures of AlpJ and ActVA-Orf6, SDS-PAGE of purified proteins, HPLC traces of prosthetic group identification in AlpG, negative controls of enzymatic reactions, SOD inhibition reactions, and HRMS spectrum of hydroquinone–kinobscurinone 10. [file Beilstein_J_Org_Chem-20-1198-s001.pdf]

BEILSTEIN JOURNAL OF ORGANIC CHEMISTRY

Supporting Information

for

Cofactor-independent C–C bond cleavage reactions catalyzed by the AlpJ family of oxygenases in atypical angucycline biosynthesis

Jinmin Gao, Liyuan Li, Shijie Shen, Guomin Ai, Bin Wang, Fang Guo, Tongjian Yang, Hui Han, Zhengren Xu, Guohui Pan and Keqiang Fan

Beilstein J. Org. Chem. **2024,** *20,* 1198–1206. doi:10.3762/bjoc.20.102

Sequence comparison results and phylogenetic tree of AlpJ-family enzymes and anthrone oxygenases, crystal structures of AlpJ and ActVA-Orf6, SDS-PAGE of purified proteins, HPLC traces of prosthetic group identification in AlpG, negative controls of enzymatic reactions, SOD inhibition reactions, and HRMS spectrum of hydroquinone–kinobscurinone 10

License and Terms: This is a supporting information file under the terms of the Creative Commons Attribution License (https://creativecommons.org/licenses/by/4.0). Please note that the reuse, redistribution and reproduction in particular requires that the author(s) and source are credited and that individual graphics may be subject to special legal provisions.
The license is subject to the *Beilstein Journal of Organic Chemistry* terms and conditions: (https://www.beilstein-journals.org/bjoc/terms)

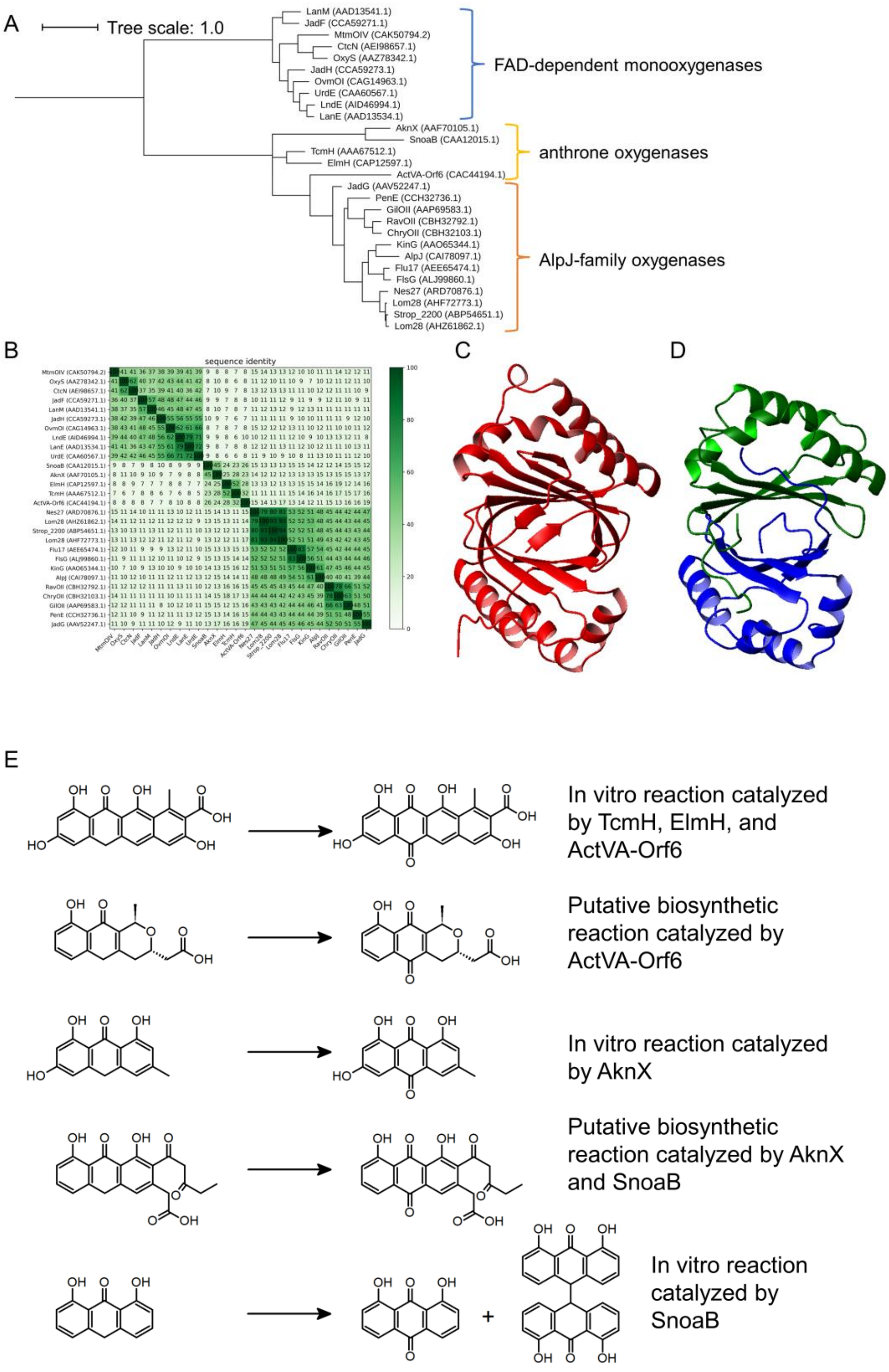

Figure S1: Sequence and structure comparison of AlpJ-family oxygenases and anthrone oxygenases. (A) Phylogenetic tree of AlpJ-family oxygenases and anthrone oxygenases, with several FAD-dependent monooxygenases as outgroups. The

GenBank Accession Numbers are listed in brackets. The phylogenetic tree was generated by MEGA 11 [1] using the maximum likelihood method and the Whelan and Goldman model. (B) Pairwise sequence identities of AlpJ-family oxygenases and anthrone oxygenases calculated by parasail_aligner [2]. (C) Protein structure of AlpJ monomer (PDB: 5F9P) [3]. (D) Protein structure of ActVA-Orf6 dimer (PDB: 1LQ9) [4]. The two monomers are colored in green and blue, respectively. (E) Reactions catalyzed by anthrone oxygenases [5-12].

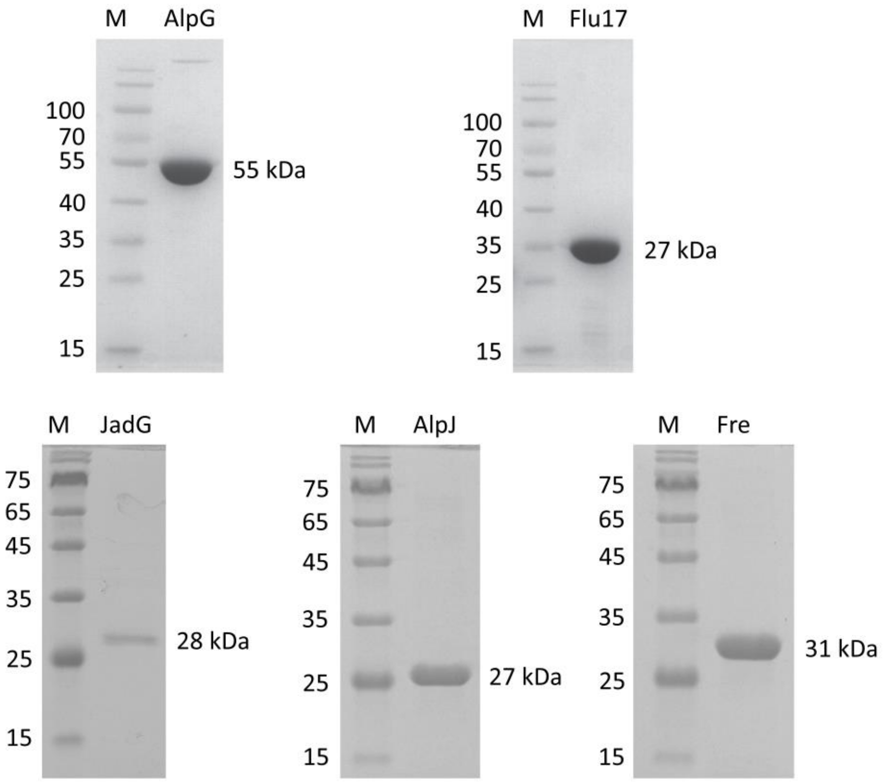

Figure S2: SDS-PAGE of purified proteins AlpG, Flu17, JadG, AlpJ, and Fre. Lane M: protein marker.

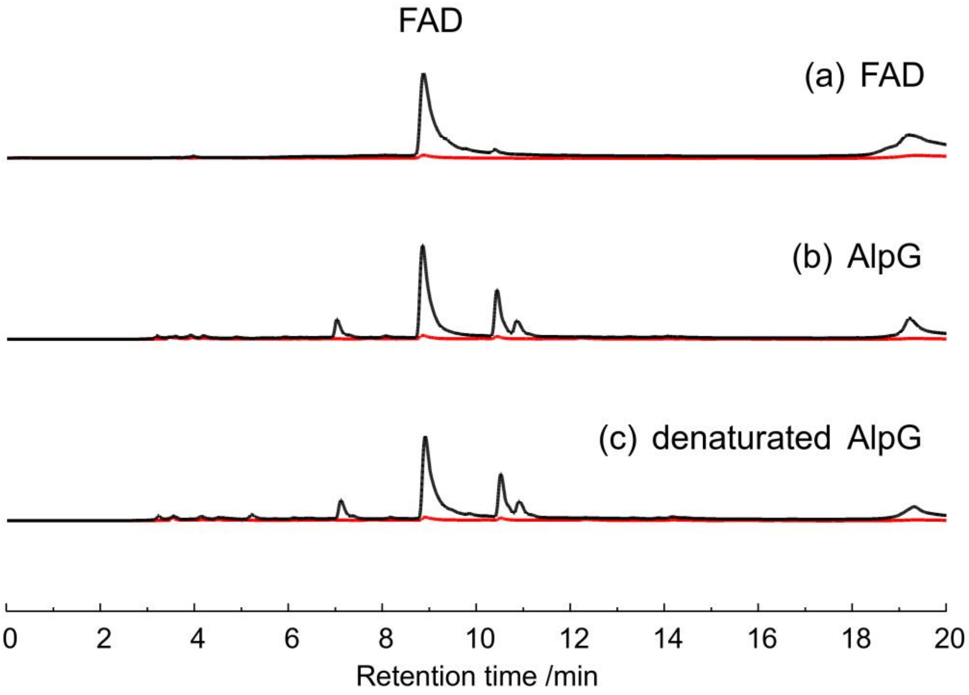

Figure S3: HPLC traces of (a) FAD standard, (b) purified AlpG protein, and (c) supernatant of the denatured AlpG. UV absorption at 266 nm (black line) and 313 nm (red line) is displayed for each reaction.

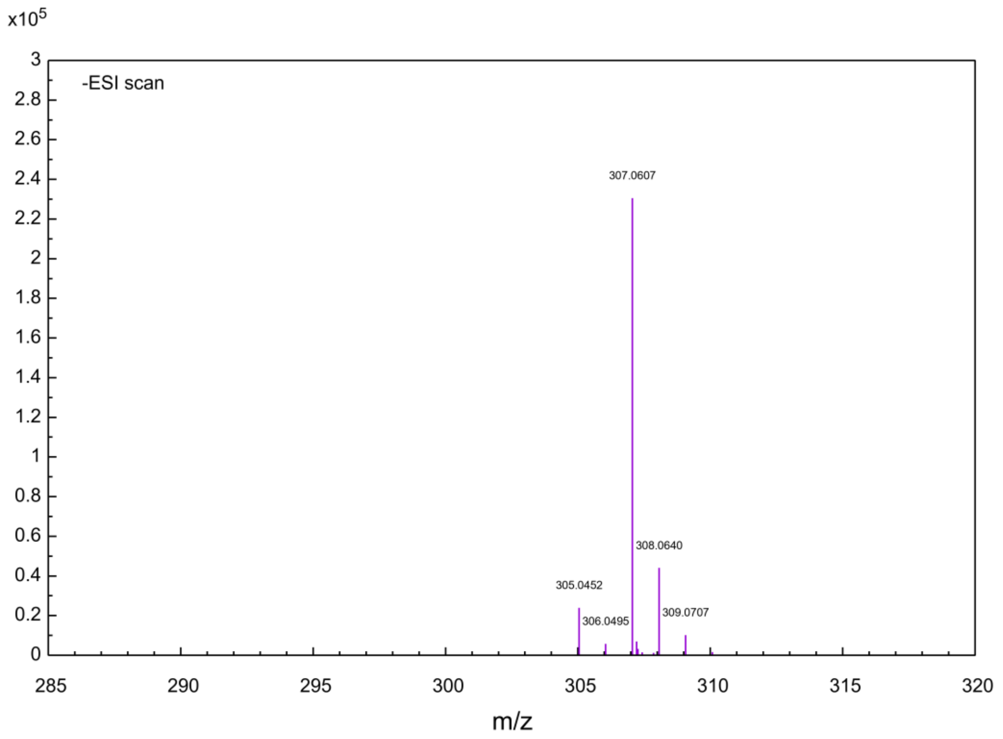

Figure S4: HRMS spectrum of hydroquinone–kinobscurinone **10**.

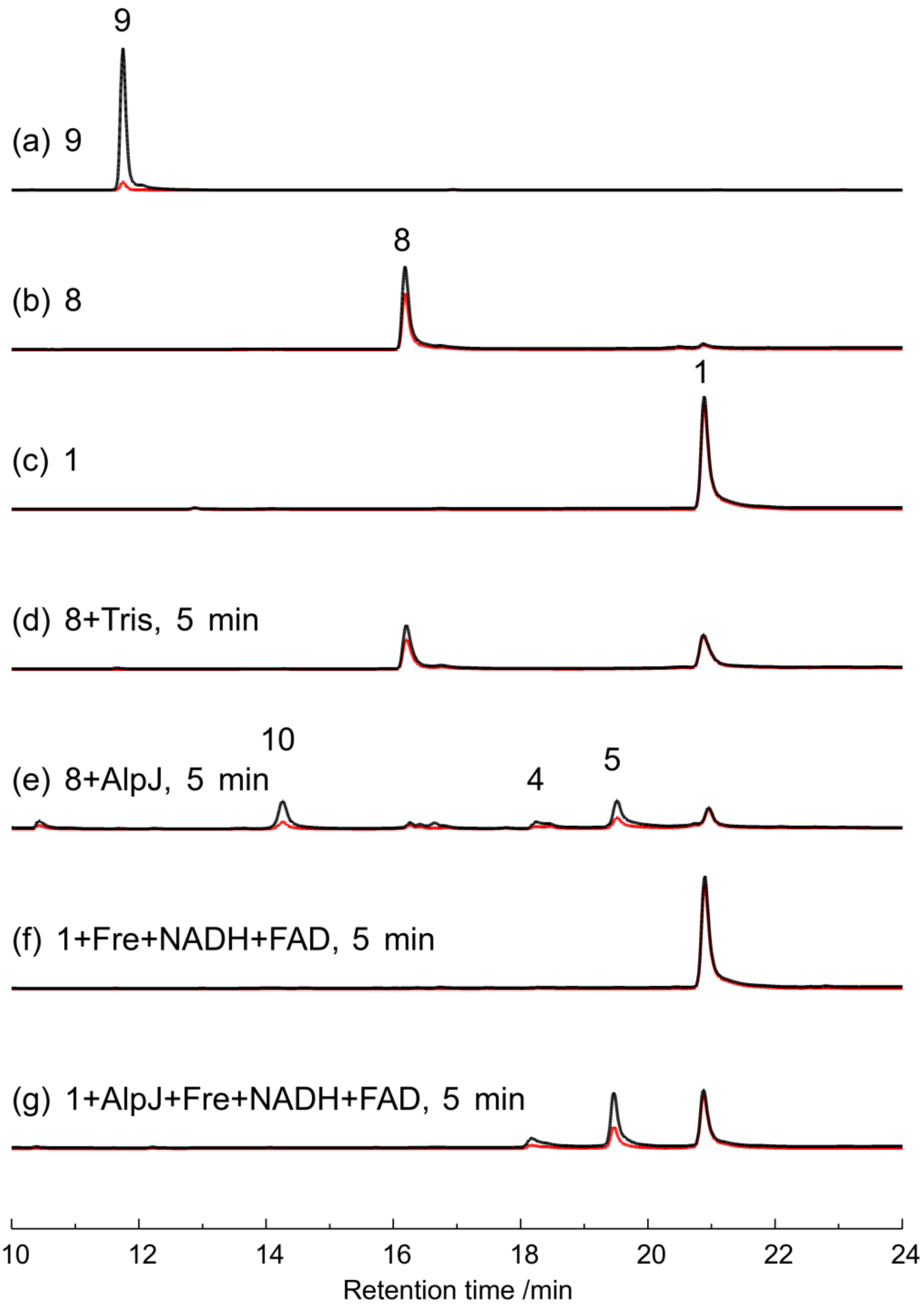

Figure S5: HPLC traces of negative controls. UV absorption at 266 nm (black line) and 313 nm (red line) is displayed for each reaction.

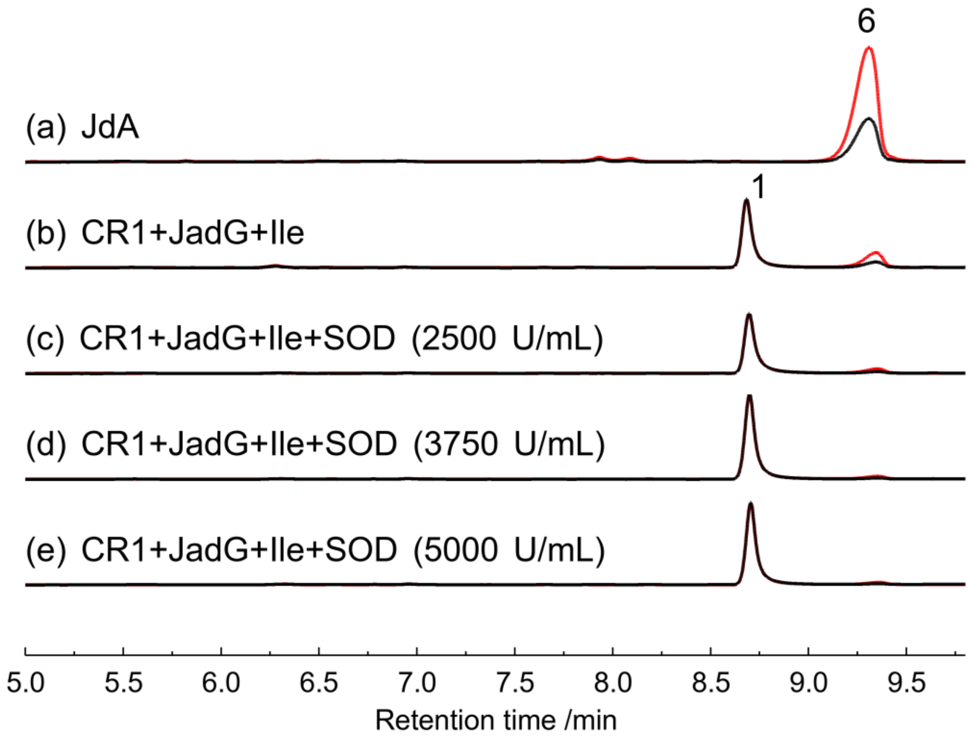

Figure S6: HPLC analysis of the effects of adding different doses of SOD to reactions of CR1 catalyzed by JadG. UV absorption at 266 nm (black line) and 313 nm (red line) is displayed for each reaction.

References

1. Tamura, K.; Stecher, G.; Kumar, S. *Molecular biology and evolution* **2021**, *38* (7), 3022-3027.
2. Daily, J. *BMC bioinformatics* **2016**, *17*, 81.
3. Pan, G.; Gao, X.; Fan, K.; Liu, J.; Meng, B.; Gao, J.; Wang, B.; Zhang, C.; Han, H.; Ai, G.; Chen, Y.; Wu, D.; Liu, Z.-J.; Yang, K. *ACS chemical biology* **2017**, *12* (1), 142-152.
4. Sciara, G.; Kendrew, S. G.; Miele, A. E.; Marsh, N. G.; Federici, L.; Malatesta, F.; Schimperna, G.; Savino, C.; Vallone, B. *The EMBO journal* **2003**, *22* (2), 205-15.
5. Kendrew, S. G.; Hopwood, D. A.; Marsh, E. N. *Journal of bacteriology* **1997**, *179* (13), 4305-10.
6. Li, X.; Li, X.; Zhang, Q.; Lv, P.; Jia, Y.; Wei, D. *Organic & biomolecular chemistry* **2022**, *20* (28), 5525-5534.
7. Shen, B.; Hutchinson, C. R. *Biochemistry* **1993**, *32* (26), 6656-63.
8. Chung, J.-y.; Fujii, I.; Harada, S.; Sankawa, U.; Ebizuka, Y. *Journal of bacteriology* **2002**, *184*, 6115-6122.
9. Rafanan, E. R., Jr.; Le, L.; Zhao, L.; Decker, H.; Shen, B. *Journal of natural products* **2001**, *64* (4), 444-9.
10. Grocholski, T.; Koskiniemi, H.; Lindqvist, Y.; Mantsala, P.; Niemi, J.; Schneider, G. *Biochemistry* **2010**, *49* (5), 934-44.
11. Machovina, M. M.; Usselman, R. J.; DuBois, J. L. *The Journal of biological chemistry* **2016**, *291* (34), 17816-28.
12. Machovina, M. M.; Ellis, E. S.; Carney, T. J.; Brushett, F. R.; DuBois, J. L. *The Journal of biological chemistry* **2019**, *294* (10), 3661-3669.
